# Automated High-Order Shimming for Neuroimaging Studies

**DOI:** 10.3390/tomography9060168

**Published:** 2023-12-01

**Authors:** Jia Xu, Baolian Yang, Douglas Kelley, Vincent A. Magnotta

**Affiliations:** 1 Department of Radiology, University of Iowa, Iowa City, IA 52242, USA; 2 GE Healthcare, Waukesha, WI 53188, USA; baolian.yang@ge.com; 3 GE Corporate Consultant, Contracted through Kelly Services, Fairfax, CA 94930, USA; douglas.kelley@ge.com; 4 Department of Psychiatry, University of Iowa, Iowa City, IA 52242, USA; 5 Department of Biomedical Engineering, University of Iowa, Iowa City, IA 52242, USA

**Keywords:** high-order shimming, B_0_ inhomogeneity, fMRI, magnetic resonance spectroscopy, MRS, EPI, spectral lineshape, image quality

## Abstract

B_0_ inhomogeneity presents a significant challenge in MRI and MR spectroscopy, particularly at high-field strengths, leading to image distortion, signal loss, and spectral broadening. Existing high-order shimming methods can alleviate these issues but often require time-consuming and subjective manual selection of regions of interest (ROIs). To address this, we proposed an automated high-order shimming (autoHOS) method, incorporating deep-learning-based brain extraction and image-based high-order shimming. This approach performs automated real-time brain extraction to define the ROI of the field map to be used in the shimming algorithm. The shimming performance of autoHOS was assessed through in vivo echo-planar imaging (EPI) and spectroscopic studies at both 3T and 7T field strengths. AutoHOS outperforms linear shimming and manual high-order shimming, enhancing both the image and spectral quality by reducing the EPI image distortion and narrowing the MRS spectral lineshapes. Therefore, autoHOS demonstrated a significant improvement in correcting B_0_ inhomogeneity while eliminating the need for additional user interaction.

## 1. Introduction

Magnetic field (B_0_) inhomogeneity in magnetic resonance imaging (MRI) can cause unwanted signal loss, image distortion, and spectral line broadening. Robust and automated shimming procedures are fundamental to the success of MRI techniques sensitive to B_0_ inhomogeneity, such as magnetic resonance spectroscopy (MRS) [1,2] and echo-planar imaging (EPI) [3].

B_0_ shimming is a procedure that the MRI scanner performs to minimize the B_0_ variation, thereby enhancing image quality. Specifically, based on the measured B_0_ field maps, i.e., the B_0_ field distribution within the region of interest (ROI), MRI hardware shim coils generate additional B_0_ fields to compensate for these inhomogeneities. There are mainly two classes of automatic shimming techniques: projection-based shimming and image-based shimming [1,4]. Projection-based shimming methods, such as the Fast Automatic Shimming Technique by Mapping Along Projections (FASTMAP) [5,6,7], utilize fields measured along six 1D column projections to calculate the first- and second-order spherical harmonic (SH) shim currents. While projection-based shimming has been proven to be fast and efficient for small-volume applications such as single-voxel MRS, its selected column projections may not capture localized field inhomogeneities [7]. Image-based shimming methods acquire 3D field maps over a ROI and hence are more suitable for arbitrarily shaped volumes such as human brains [8,9].

High-order shimming methods employ higher-order spherical harmonics to model the B_0_ inhomogeneity, allowing for a more accurate B_0_ inhomogeneity correction and improved image quality. The introduction of high-order shims confers significant benefits to both brain and body imaging, especially at high-field strengths of 3T and above, by mitigating increased field inhomogeneity. For example, the High Order Shimming (HOS) software on the GE Signa 7.0T scanner calculates all shimming currents up to third order to minimize the B_0_ inhomogeneity within a selected ROI of the acquired gradient-echo-based field maps [8]. However, to date, most high-order shimming methods need a manually defined shim ROI if different from the imaging volume, which introduces intra- and inter-operator variability. Various automated brain extraction algorithms have emerged to address such variabilities, removing non-brain tissues from MR images, including the B_0_ field map [10,11]. In addition, skull-stripping by brain extraction can reduce the field magnitude variations caused by the presence of extracranial lipid signals [1,12]. However, using the extracted brain as the shimming ROI is typically not recommended since brain extraction is time-consuming and may not be robust when applied to lower resolution data [1]. Recently, the development of deep-learning-based image segmentation tools now enables fast and robust brain extraction [13,14]. Among the existing (semi-)automated brain extraction algorithms, the deep-learning-based HD-BET stands out, as it can generate brain masks from diverse MRI scans, even in the presence of pathology, with a high degree of accuracy [10]. Based on a 3D U-net architecture, HD-BET outperforms conventional and 2D deep-learning (DL) brain extraction tools, albeit at a higher computational cost [13,14]. However, modern GPUs enable the runtime of HD-BET to be reduced to less than one minute [10].

In this work, we propose an automated High Order Shimming (autoHOS) prototype to conduct objective and automated high-order shimming based on automated brain extraction. Our method employs a combination of automated brain extraction [10] and image-based high-order shimming [8] to automatically define the shim ROI and compute the shim currents. We assessed our method using in vivo echo-planar imaging and spectroscopic studies performed on 3T and 7T scanners. Our findings indicate that autoHOS markedly improves the B_0_ homogeneity, leading to superior image and spectral quality.

## 2. Materials and Methods

### 2.1. Automated HOS Pipeline and Implementation

**Figure 1:**
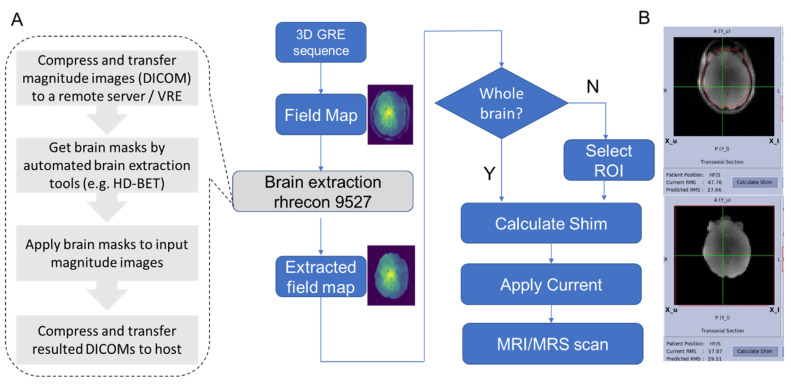
Flowchart of automated HOS: (**A**) First, a 3D gradient-echo pulse sequence is acquired to generate a B_0_ field map. Subsequentially, the DL-based brain extraction software operates on the image reconstruction computer or a remote server to produce a skull-stripped field map. By default, the brain-extracted field map is used for least-squares optimization of shim currents without ROI selection. (**B**) The top image shows an ROI (red) drawn manually for the brain region to be used for shim current optimization, while the bottom image shows the brain region automatically extracted also used for the same purpose.

The autoHOS prototype is developed in Python 3.8 and Tcl/Tk and depends on HD-BET and GE (GE HealthCare, Waukesha, WI, USA) HOS software. Figure 1A shows the flowchart of automated HOS. First, a 3D field map covering the whole brain (e.g., 28.0 × 28.0 × 21.0 cm^3^ box) with 128 × 128 × 64 matrix size is acquired using a 3D gradient-echo pulse sequence. The magnitude images of the 3D field map are transferred to Volume Recon Engine (VRE), the image reconstruction architecture of GE MRI scanners, for automated brain extraction using a GPU. Alternatively, the magnitude images can be transferred to a remote computer with a GPU if one is not available on the VRE. Brain masks are generated by HD-BET in GPU mode and applied to magnitude images of the field map. The obtained skull-stripped magnitude images are then transferred back to the MRI host computer for least-squares calculation of the updated shim currents. By default, the autoHOS procedure will automatically adjust the high order shims over the whole brain (Figure 1B). However, it also supports selection of smaller ROIs based on voxel placement for single-voxel MRS applications (Figure S1).

### 2.2. Participants and Experimental Protocol

Eleven participants were recruited into this study after receiving informed written consent using an Institutional Review Board (IRB) protocol. The subjects had an average age of 51 ± 22 years and an average weight of 68 ± 13 kg. The image quality of single-shot gradient echo EPI images was used to benchmark the performance of the linear shimming, HOS, and autoHOS at both 3T and 7T. In addition, the spectral linewidth of ^31^P MRS/MRSI at 7T was used to compare the shimming performance of HOS and autoHOS.

### 2.3. 3T MR Imaging Protocol

Images of the participants were acquired on a GE SIGNA Premier 3.0T MRI system equipped with a 48-channel head coil. For each subject, 3D sagittal T2-weighted CUBE sequences were obtained with parameters: TE = 92 ms, TR = 3202 ms, FOV = 256 × 256 × 196 mm^3^, matrix = 256 × 256 × 196, flip = 90⁰, and bandwidth = 488 Hz/pixel. Additionally, 2D T2*-weighted single-shot gradient echo EPI images consisting of 35 slices were acquired with TE = 30 ms, TR = 2000 ms, FOV = 220 × 220 × 140 mm^3^, matrix = 64 × 64 × 35, flip = 80⁰, pixel spacing= 3.4375 × 3.4375 mm^2^, bandwidth = 977 Hz/pixel, and it encompassed 10 temporal points. EPI images were acquired using linear shimming, HOS, and autoHOS settings, respectively. The B_0_ field map was measured using phase difference images using a multi-echo GRE sequence with TEs of 4.5 and 6.8 ms. The EPI images and B_0_ field map images were acquired after performing each shimming technique (linear, manual HOS, and autoHOS).

### 2.4. 7T MR Imaging Protocol

7T images were acquired on a GE SIGNA 7.0T scanner equipped with a Nova Medical 2-channel transmit/32-channel receive head coil. For each subject, 3D sagittal T2-weighted CUBE sequences were obtained with parameters: TE = 66 ms, TR = 3000 ms, FOV = 198 × 198 × 212 mm^3^, matrix = 220 × 220 × 212, flip = 90°, pixel spacing = 0.8594 × 0.8594 mm^2^, and slice thickness = 1 mm. Additionally, 2D T2*-weighted single-shot gradient echo EPI images consisting of 50 slices were acquired with TE = 20 ms, TR = 2000 ms, FOV = 198 × 198 × 150 mm^3^, matrix = 110 × 110 × 50, flip = 75⁰, pixel spacing = 1.7188 × 1.7188 mm^2^, and it encompassed 20 temporal points. Similar to 3T, EPI images were also acquired using linear shimming, HOS, and autoHOS settings, respectively. The B_0_ field map was measured using the same strategy as employed for the 3T, with TEs of 2.0 and 2.9 ms. The EPI images and B_0_ field map images were acquired after performing each shimming technique.

MRS data were collected on the GE SIGNA 7.0T scanner using a dual-tuned RAPID Biomedical ^31^P/1H coil. The nonlocalized ^31^P were acquired using a free induction decay (FID) sequence with the following parameters (TR = 2000 ms, flip angle = 20°, spectral bandwidth = 10,000 Hz, # samples = 2048, FOV = 160 × 160 × 100 mm^3^). The ^31^P MRSI was acquired with an FID chemical shift imaging (CSI) sequence in cerebellum using the following parameters: (TR = 2000 ms, flip angle = 45°, spectral bandwidth = 10,000 Hz, # samples = 2048, FOV = 160 × 160 × 100 mm^3^, matrix size = 4 × 4 × 1). ^31^P MRS data were acquired using both the standard manual HOS approach as well as the proposed autoHOS approach.

### 2.5. Image Analysis

Three-dimensional T2-weighted anatomical images were downsampled to match the spatial resolution of the T2*-weighted single-shot gradient echo EPI images using AFNI and served as an anatomical reference assumed to be free of image distortion [15]. All T2*-weighted EPI images and B_0_ field maps were aligned to these T2-weighted reference images using rigid-body transformation through the ANTs registration tool [16]. Brain extraction of all images was performed using HD-BET [10]. The edges of the skull-stripped images were obtained using AFNI’s 3dEdge command. To evaluate the efficiency of different shimming strategies, the standard deviation of B_0_ values was estimated both in each slice and across the whole field map, serving as an indicator of field homogeneity, respectively. The structural similarity index (SSIM) and peak signal-to-noise ratio (PSNR) were calculated using scikit-image to compare the image quality of the EPI images to the reference scans acquired with different shimming strategies [17].

The ^31^P MRS/MRSI data were processed and analyzed using in-house Python scripts. For nonlocalized ^31^P MRS, the obtained FIDs were zero-filled to 8K and apodized with a 5 Hz line-broadening before Fourier transformation. Nmrglue was used to conduct peak picking of the phase-corrected absorptive spectrum and quantify the width of the picked peaks as full width at half maximum (FWHM) [18]. For 2D ^31^P MRSI, a 20 Hz Gaussian line-broadening and a Hamming filter were applied to both the spectral and spatial dimensions of the k-space data prior to Fourier transformation. Because the ^31^P MRSI spectrum cannot be phased due to its prolonged dead time, AMARES was used to quantify the linewidth as damping factors [19]. 

The Wilcoxon paired-sum test was used to compare the performance of various shimming methods.

## 3. Results

### 3.1. Comparison of B_0_ Homogeneities by Shimming Techniques

**Figure 2:**
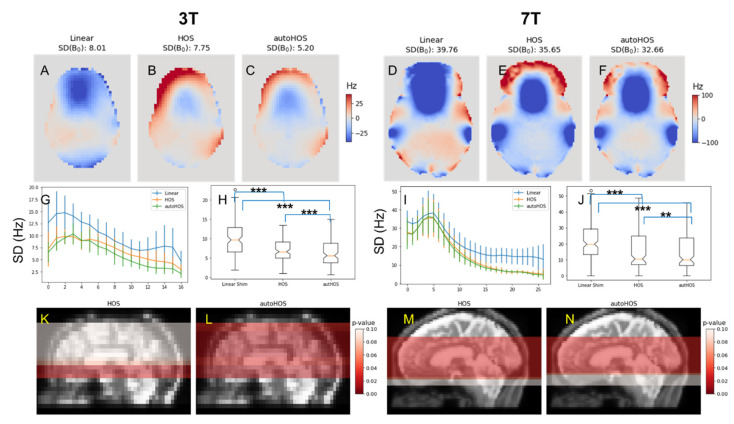
Comparison of MR shimming approaches on the resulting field map. Linear, high order shimming (HOS), and automated HOS (autoHOS). AutoHOS and HOS improve global B_0_ homogeneity. (**A**–**C**) and (**D**–**F**) show representative B_0_ field maps at 3T and 7T, respectively. (**G**) and (**I**) display SD(B_0_) values across slices at 3T and 7T. (**H**) and (**J**) present boxplots of the global SD(B_0_) comparisons, where ** indicates *p* < 0.01 and *** means *p* < 0.001. (**K**,**L**) and (**M**,**N**) highlighted in red where HOS and autoHOS show significant improved shimming (*p* < 0.05) with results shown on the corresponding sagittal MRI images for 3T and 7T, respectively.

The standard deviation (SD) of the field map acquired after shimming within the brain region was used to assess the B_0_ homogeneity (Figure 2) for each of the methods. Overall, the HOS and autoHOS displayed a similar performance at both 3T and 7T. Among the seven subjects studied, both high order shimming methods consistently demonstrated reduced smaller B_0_ standard deviation values across slices as compared to linear shimming, with most of these reductions reaching statistical significance (*p* < 0.05) as highlighted in red on the sagittal view in Figure 2K–N. At 3T, HOS’s reduction in B_0_ inhomogeneity was significant only in tissues bordering the sinuses and ear canals, while autoHOS showed significant reductions across most slices. In contrast, at 7T, while both autoHOS and HOS displayed a comparable number of significantly improved slices (Figure 2M,N), when comparing the global SD(B_0_) of HOS and autoHOS with linear shimming, notable distinctions were observed (Figure 2H,J), with both HOS and autoHOS significantly outperforming linear shimming (*p* < 0.001). Furthermore, autoHOS was superior to HOS at both 3T (*p* < 0.001) and 7T (*p* < 0.01).

### 3.2. Comparison of EPI Distortion by Shimming Techniques

In addition to assessing the field map, we evaluated the performance of various shimming methods by comparing the quality of the single-shot gradient-echo EPI image acquired using the three shimming approaches. Specifically, we calculated the PSNR and SSIM between the slices of the acquired T2*-weighted gradient-echo EPI images and the corresponding slices of the reference anatomical T2-weighted CUBE images. 

**Figure 3:**
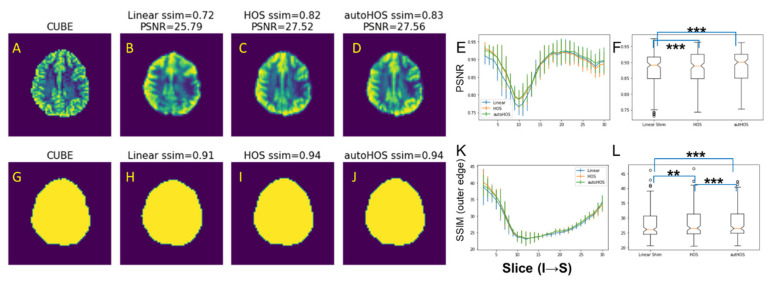
HOS and autoHOS reduce EPI image distortions at 3T. (**A**–**D**) and (**G**–**J**) display representative slices of the reference T2-weighted CUBE images (**A**,**G**), EPI images acquired with linear shimming (**B**,**H**), HOS (**C**,**I**), and autoHOS (**D**,**J**). The top row (**A**–**D**) and bottom row (**G**–**J**) showcase the EPI images and the corresponding outer edge of the brain in the EPI images, respectively. Panels (**E**) and (**K**) illustrate the PSNR and SSIM of the outer brain contour, respectively. Panels (**F**) and (**L**) depict comparisons of global PSNR and SSIM of outer brain contour, respectively. (** *p* < 0.01, *** *p* < 0.001).

At 3T, EPI image quality acquired with different shimming methods was compared using PSNR (Figure 3A–F). Both HOS and autoHOS exhibited modest increases in PSNR values across slices over the brain (Figure 3E), and on a global scale, both methods showed significant superiority over linear shimming (*p* < 0.001, Figure 3F). 

The SSIM of the edge images was used to gauge the underlying distortions in the acquired EPI images as compared to the corresponding slice acquired using a standard T2 acquisition (Figure 3G–L). Similar to PSNR comparison, both HOS and autoHOS displayed slight improvements in SSIM across slices (Figure 3K). Notably, although both HOS and autoHOS techniques significantly improved the SSIM values (*p* < 0.01 for HOS, *p* < 0.001 for autoHOS), autoHOS outperformed HOS (*p* < 0.001, Figure 3L).

**Figure 4:**
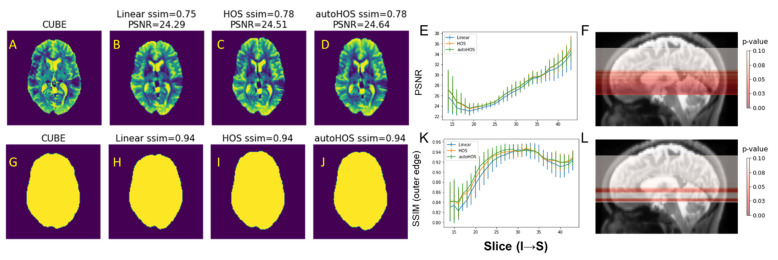
HOS and autoHOS reduce EPI image distortions at 7T. (**A**–**D**) and (**G**–**J**) display representative slices of the reference T2-weighted CUBE images (**A**,**G**), EPI images acquired with linear shimming (**B**,**H**), HOS (**C**,**I**), and autoHOS (**D**,**J**). The top row (**A**–**D**) and bottom row (**G**–**J**) showcase the EPI images and the corresponding outer edge of the brain, respectively. Panels (**E**) and (**K**) illustrate the PSNR and SSIM of the outer brain contour, respectively. Panels (**F**) and (**L**) highlight slices in which both HOS and autoHOS demonstrate significant improvement, marked in red on sagittal view anatomical MRI, for PSNR and SSIM of the outer brain contour, respectively.

Following our analyses at 3T, similar assessments were conducted at 7T. The overall findings closely mirrored those observed at the lower field strength. Overall, both HOS and autoHOS demonstrated statistically significant improvements over linear shimming (*p* < 0.001). However, between these two high-order shimming methods, no noticeable differences were observed (Figure S2A), except for the SSIM index for the outer brain contour, where autoHOS modestly but significantly outperformed HOS (Figure S2B). In a slice-by-slice comparison, EPI images obtained using HOS and autoHOS exhibited higher PSNR and SSIM values than those acquired with linear shimming across the entire brain (Figure 4E,K). Importantly, slices including regions close to air cavities, such as the frontal lobes and ear canals, exhibited significant improvements with both HOS and autoHOS (*p* < 0.05, highlighted in red in Figure 4F,L; also, see representative examples in Figure S3).

### 3.3. Superiority of autoHOS in MRS Spectral Linewidths

**Figure 5:**
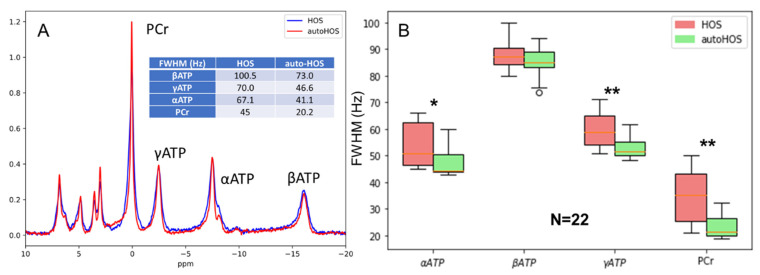
AutoHOS narrows the spectral linewidth of nonlocalized ^31^P MRS. (**A**) Full Width at Half Maximum (FWHM) comparison between HOS and autoHOS at 7T, illustrated by a representative case. (**B**) Boxplot representing the FWHM of bioenergetic ^31^P MRS peaks (*n* = 22) *: *p* < 0.05, **: *p* < 0.01.

The MRS spectral linewidth serves as a sensitive indicator of B_0_ homogeneity within the selected MRS voxel. We chose the spectral peak width of bioenergetic peaks that are distinctly separated in ^31^P MRS, including ATPs and phosphocreatine (PCr), to assess the shimming performance. The comparison between MRS lineshapes acquired with HOS and autoHOS underscored the distinctive advantage of autoHOS in MRS. This advantage is exemplified by the narrower linewidth of representative cases of both nonlocalized ^31^P MRS (Figure 5A) and ^31^P MRSI (Figure S4) at 7T. In 22 nonlocalized ^31^P MRS sessions, autoHOS significantly reduced the peak width of PCr, αATP, and γATP (Figure 5B). Interestingly, even though the linewidths for βATP were narrower in spectra acquired with autoHOS, the difference did not achieve statistical significance. 

## 4. Discussion

B_0_ inhomogeneity has always been a challenge in MRI, particularly at higher magnetic field strengths like 7T. It introduces artifacts, reduces the SNR, and hampers the accuracy of quantitative measurements. The significance of the issue marks the importance of developing advanced shimming methods such as autoHOS. While both HOS and autoHOS significantly reduce B_0_ inhomogeneity, autoHOS outperformed HOS on a broader scale. First, while not quantified, autoHOS is inherently faster and more objective than HOS because it eliminates the need for manual ROI selection via GUI interaction. In addition, the automated tool provides similar or better results as compared to the manual approach for high order shimming and is not biased based on the technologist running the scan. Compared to projection-based shimming techniques, such as FASTMAP and its derivatives [5,6,7,20], autoHOS does not rely on small spherical or cubic ROIs. Thus, it is more suitable for applications involving larger and often irregularly-shaped ROIs, such as EPI images, MRSI, and non-localized MRS. However, the limited number of field map points within a smaller spectroscopic voxel may affect the effectiveness of image-based shimming. In contrast, the 1D nature of projection-based shimming allows extended encoding times, which enhances the sensitivity to frequency variations and maximizes the accuracy of the magnetic field maps within such a small voxel. Although projection-based shimming has proven be to more efficient than full 3D B_0_ field mapping for single-voxel MRS [1,7], the new rapid 3D B_0_ field mapping techniques on which autoHOS is based are comparably fast and can easily incorporate third- or higher-order shimming terms. For instance, on a GE 7.0T SIGNA scanner, acquiring the B_0_ field map by a 3D gradient-echo pulse sequence with a matrix of 128 × 128 × 64 takes only 43 s. Immediately following the acquisition of the B_0_ field map, the brain extraction takes less than 30 s, thanks to the SIGNA VRE equipped with the NVIDIA T4 Tensor Core GPU. As a result, the entire autoHOS procedure can be completed within 2 min. 

### 4.1. High-Order Shimming Techniques Improve B_0_ Homogeneity Similarly at 3T and 7T

For B_0_ field maps at both 3T and 7T, the standard deviation of B_0_ values (SD(B_0_)) indicates that high-order shimming techniques, both manual HOS and autoHOS, significantly reduce B_0_ inhomogeneity across the entire brain at both 3T and 7T. As depicted in Figure 2G–J, the magnitude of SD(B_0_) of 7T is considerably larger than that at 3T, suggesting the increased inhomogeneity of ultra-high field strength 7T. Globally, HOS outperforms linear shimming significantly at both 3T and 7T (Figure 2H). A slice-by-slice comparison reveals that HOS substantially outperforms linear shimming in several slices adjacent to air cavities (Figure 2K), suggesting that the inclusion of the extracranial lipid pixels particularly affect the low-resolution field map (64 × 64) at 3T and introduce errors. While for 7T, HOS demonstrates significance in most slices (Figure 2M), suggesting the high-resolution field map (128 × 128) may alleviate the issues introduced by extracranial lipid pixels. In contrast, autoHOS, which is based on skull-stripped B_0_ field maps, shows significant improvement across most slices at both 3T and 7T (Figure 2L,N).

### 4.2. HOS and AutoHOS Reduces EPI Distortions

For EPI images, high-order shimming techniques have been shown to reduce image distortions caused by B_0_ inhomogeneity. At both 3T and 7T, although autoHOS is evidently superior to linear shimming, it does not show considerable improvement compared to HOS when gauging image quality with PSNR. This could be attributed to the brain extraction process, which does not remove all pixels with possible phase offsets [1], although the erroneous extracranial lipid pixels are already stripped. Remarkably, autoHOS produces a modest yet significantly improved brain outer edge compared to HOS at both 3T and 7T (Figure 3L and Figure S2B). This improvement can enhance fMRI studies by better co-registering fMRI images with structural MRI and facilitating region-of-interest analyses. 

### 4.3. AutoHOS Significantly Improves Nonlocalized ^31^P MRS

For nonlocalized ^31^P MRS at 7T, the benefits of autoHOS become particularly evident. AutoHOS substantially narrows the lineshapes, suggesting enhanced global B_0_ homogeneity, which is further corroborated by the notably improved B_0_ maps. Such improvement is crucial as B_0_ variation is the primary hurdle in achieving accurate spectral quantification. Importantly, spectral linewidth data points of autoHOS show tighter clustering across ATP and PCr peaks, indicating superior reproducibility, a benefit that probably can also be attributed to the elimination of operator variability. An intriguing observation is that although autoHOS visibly reduces the spectral width of βATP peak (Figure 5B), this narrowing does not achieve statistical significance like γATP and PCr. This reason behind this is that when B_0_-induced line broadening (e.g., ~25 Hz as shown in Figure 5A) affects all ^31^P peaks, the relative percentage change is inherently smaller for broadened peaks like βATP than it is for the sharper peaks. 

### 4.4. Limitations

Our study still has several limitations. First, the relatively small sample size (*n* = 7) potentially limits the statistical power of our findings related to B_0_ field maps and EPI images. Second, due to constraints on scanner access and subject availability, the 22 sessions of nonlocalized MRS were acquired from six subjects over a span of 4 months—11 sessions were before and 11 after the deployment of autoHOS on our 7T scanner. Ideally, we would have compared the three shimming techniques using the same subjects within the same sessions, following the approach we took for assessing shimming performance with B_0_ field map and EPI images.

It is worth noting that although we presented an example where autoHOS narrows lineshapes in a representative MRSI voxel (Figure S4), this comparison was made between a ^31^P MRSI acquired with autoHOS and one acquired with HOS, where the entire brain was manually selected. However, it is common to select the ROI as defined by the voxel of the single-voxel or multi-voxel MRS scan. In such cases, there may be no difference between autoHOS and HOS since brain extraction is unnecessary for manually selecting a region smaller than the entire brain. Similarly, autoHOS does not impact local neuroimaging studies focusing on specific regions that do not include the skull. For this reason, the current autoHOS tool retains the ability to define the shimming ROI of interest based on the MRS prescription volume.

### 4.5. Future Work

In the future, we plan to improve the field map for shim calculation by utilizing brain masks generated as part of the automated prescription algorithm on the scanner. This would eliminate the most time-consuming aspect of the current solution. Ultimately, our goal is to integrate this into the pre-scan process, similar to coil calibration and center frequency adjustment. Furthermore, since autoHOS is deployed with Docker and is inherently cross-platform, it can be easily ported to MRI systems manufactured by vendors other than GE. 

## 5. Conclusions

To address the important challenges of B_0_ inhomogeneity, especially at high field strengths like 3T and 7T, we proposed and rigorously assessed the autoHOS method against existing linear shimming and manual HOS techniques on GE 3T and 7T scanners. Our findings underscored that both HOS and autoHOS effectively mitigate B_0_ inhomogeneities at 3T and 7T. Importantly, autoHOS not only has the advantage of eliminating manual ROI selection but also shows broader superiority, particularly in reducing EPI image distortions and narrowing MRS/MRSI spectral linewidths.

## Data Availability

The autoHOS prototype is available upon request from the corresponding author or can be accessed through the GE MR Collaboration Community (https://www.gecares.com/s/GEHCForum) via Research Community Sharing License (RCSL).

## Supplementary Materials

The following supporting information can be downloaded at: https://www.mdpi.com/article/10.3390/tomography9060168/s1, Figure S1: Selection of shim ROI.; Figure S2: HOS and autoHOS reduces EPI image distortions at 7T; Figure S3: Representative EPI images acquired with linear shimming, HOS, and autoHOS at 3T and 7T; Figure S4: Selected voxel of ^31^P MRSI lineshape comparison between HOS and autoHOS at 7T.
