# Supplementary material for: Automated High-Order Shimming for Neuroimaging Studies
Source: Tomography. 2023 Dec 1;9(6):2148–57. doi: 10.3390/tomography9060168 (PMC10748357; doi:10.3390/tomography9060168)
Supplement: Supplementary file 1 [file tomography-09-00168-s001.zip › tomography-2710072-supplementary.pdf]

SUPPORTING INFORMATION FOR

Automated High Order Shimming for Neuroimaging Studies

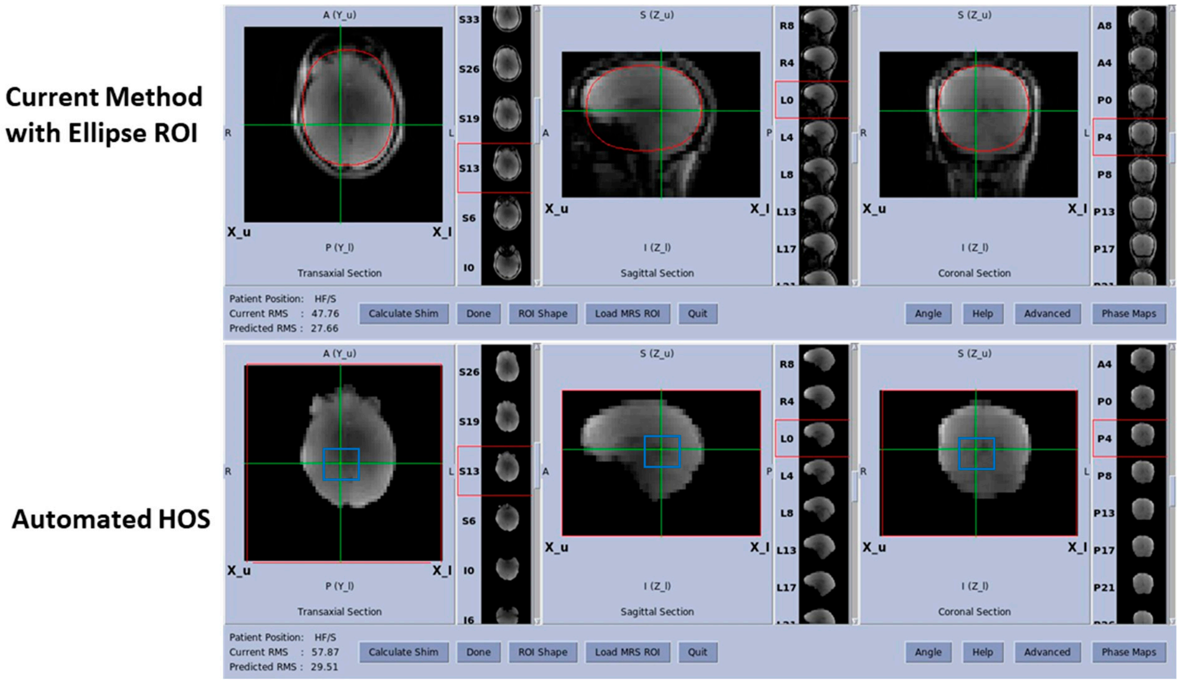

Figure S1. Selection of shim ROI. The current HOS software needs manual selection of ellipse or rectangular ROI (top). autoHOS (bottom) automatically shims the whole brain (red box). It also supports the manual selection of smaller ROI (blue box) for applications such as single-voxel MRS

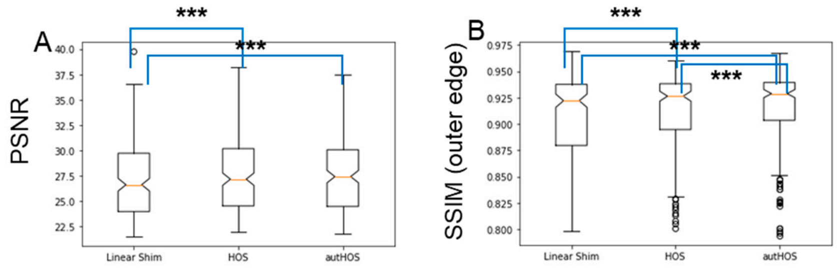

Figure S2. **HOS and autoHOS reduces EPI image distortions at 7T.** A and B illustrate comparisons of global PSNR and SSIM of outer brain contour, respectively. (** p<0.01, *** p<0.001)

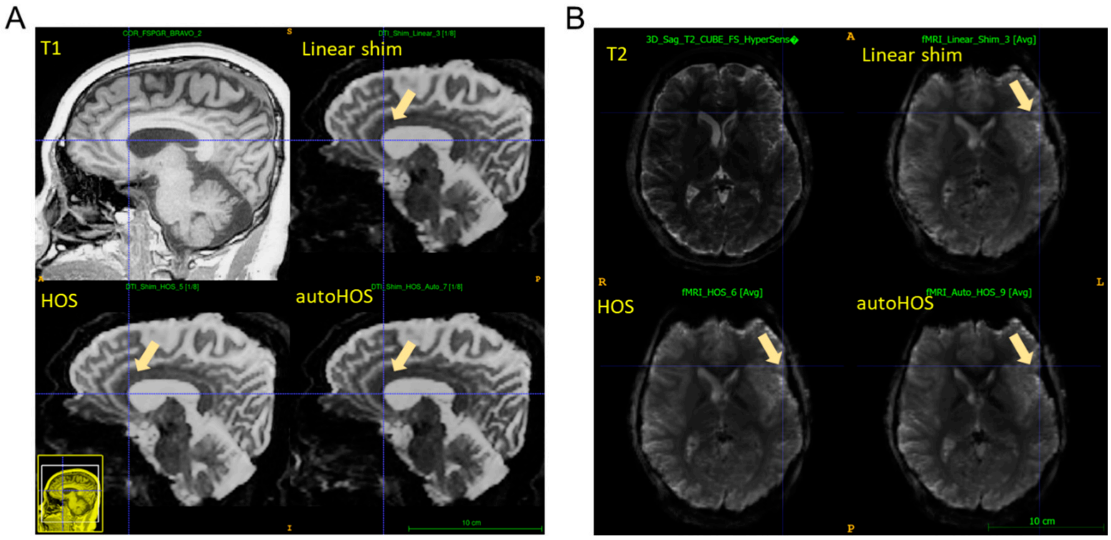

Figure S3. Representative EPI images acquired with linear shimming, HOS, and autoHOS at 3T (A) and 7T (B)

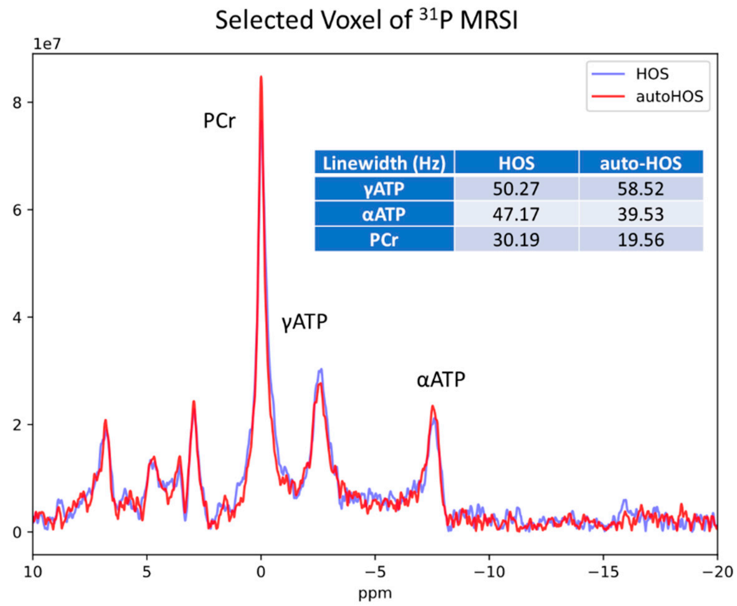

Linewidth (Hz)	HOS	auto-HOS
γATP	50.27	58.52
αATP	47.17	39.53
PCr	30.19	19.56

Figure S4. Selected voxel (8.3×8.3×8 cm^3) of ^{31}P MRSI lineshape comparison between HOS and autoHOS at 7T.
